# A Modular System for Treating Moving Anatomical Targets With Scanned Ion Beams at Multiple Facilities: Pre-Clinical Testing for Quality and Safety of Beam Delivery

**DOI:** 10.3389/fonc.2021.620388

**Published:** 2021-03-19

**Authors:** Michelle Lis, Wayne Newhauser, Marco Donetti, Moritz Wolf, Timo Steinsberger, Athena Paz, Marco Durante, Christian Graeff

**Affiliations:** ^1^ Biophysics, GSI Helmholtzzentrum für Schwerionenforschung GmbH, Darmstadt, Germany; ^2^ Department of Physics and Astronomy, Louisiana State University, Baton Rouge, LA, United States; ^3^ Department of Radiation Physics, Mary Bird Perkins Cancer Center, Baton Rouge, LA, United States; ^4^ Research and Development Department, Centro Nazionale di Androterapia Oncologia, Pavia, Italy; ^5^ Institute of Condensed Matter Physics, Technical University of Darmstadt, Darmstadt, Germany

**Keywords:** 4D therapy, carbon ion therapy, failure modes and effects analysis, motion-mitigation, patient safety, quality assurance, motion-synchronized dose delivery

## Abstract

**Background:**

Quality management and safety are integral to modern radiotherapy. New radiotherapy technologies require new consensus guidelines on quality and safety. Established analysis strategies, such as the failure modes and effects analysis (FMEA) and incident learning systems have been developed as tools to assess the safety of several types of radiation therapies. An extensive literature documents the widespread application of risk analysis methods to photon radiation therapy. Relatively little attention has been paid to performing risk analyses of nascent radiation therapy systems to treat moving tumors with scanned heavy ion beams. The purpose of this study was to apply a comprehensive safety analysis strategy to a motion-synchronized dose delivery system (M-DDS) for ion therapy.

**Methods:**

We applied a risk analysis method to new treatment planning and treatment delivery processes with scanned heavy ion beams. The processes utilize a prototype, modular dose delivery system, currently undergoing preclinical testing, that provides new capabilities for treating moving anatomy. Each step in the treatment process was listed in a process map, potential errors for each step were identified and scored using the risk probability number in an FMEA, and the possible causes of each error were described in a fault tree analysis. Solutions were identified to mitigate the risk of these errors, including permanent corrective actions, periodic quality assurance (QA) tests, and patient specific QA (PSQA) tests. Each solution was tested experimentally.

**Results:**

The analysis revealed 58 potential errors that could compromise beam delivery quality or safety. Each of the 14 binary (pass-or-fail) tests passed. Each of the nine QA and four PSQA tests were within anticipated clinical specifications. The modular M-DDS was modified accordingly, and was found to function at two centers.

**Conclusion:**

We have applied a comprehensive risk analysis strategy to the M-DDS and shown that it is a clinically viable motion mitigation strategy. The described strategy can be utilized at any ion therapy center that operates with the modular M-DDS. The approach can also be adapted for use at other facilities and can be combined with existing safety analysis systems.

## Introduction

Quality, safety, and radioprotection are integral parts of radiotherapy facilities (1). Radioprotection and area monitoring systems are designed to protect staff under the principles of justification, optimization and limitation (2). During regular operation, the critical safety operations of each accelerator are regulated by the main treatment control room and, for redundancy, by independent safety interlock systems (3). Medical systems used for radiotherapy, including accelerators, treatment control systems, and safety systems, typically take into account safety considerations during the design, construction, preclinical, and clinical phases. In addition, safety is considered in regulatory processes, e.g., in the USA, the 510k premarket clearance by the Food and Drug Administration (FDA) (21 C.F.R. § 807.81–807.97). Once in clinical use, exhaustive safety, and quality assurance testing must be periodically performed. Quality management has been an integral part of modern radiotherapy and is essential for safe and effective treatments. Organizations such as the American Association of Physicists in Medicine (AAPM), American College of Radiology (ACR), the American Society for Radiation Oncology (ASTRO), the International Atomic Energy Agency (IAEA), and the European Society for Radiology and Oncology (4) have established safety standards and guidelines (5). Radiotherapy device manufacturers and therapy centers typically agree upon test procedures as part of the acceptance testing process and guidelines are published for verifying the performance and safety of radiotherapy equipment during commissioning and periodic quality assurance testing (6–10). Beam commissioning and QA standards have been established for proton beams in the AAPM Task Group 224 report (11) and are being established for ion beam therapies through the PAR-13-371 National Cancer Institute (NCI) grant (12). As the complexity of modern treatment planning and delivery technologies, such as scanned ion beam therapy, has increased, additional consideration of safety is necessary. There is typically a lag between implementing modern therapies into the clinic and developing consensus safety guidelines for these therapies. The AAPM’s Task Group 100 (TG-100) wrote a report (13) on an analysis methodology that aims to reduce this lag. The report is a framework to prospectively assess all aspects of workflow for possible critical safety errors in existing and new therapy methods (13, 14).

Broadly, the methods of safety analysis and risk mitigation are mature, rich, and generally applicable. Several major analysis strategies have been applied to clinical radiotherapy. For example, the AAPM and the Joint Commission on Accreditation of Healthcare Organizations (JCAHO) recommended the failure modes and effects analysis (FMEA), adapted from aviation safety to radiotherapy, as a general guidance for performing safety analyses (15). Additionally, guidelines have been developed by groups such as the “Accidental and unintended exposures in radiotherapy” (ACCIRAD) project of the European Commission (EC), the Radiation Oncology Incident Learning System (RO-ILS; ASTRO, Arlington, VA), which are based on pooled data on reported adverse events (16, 17). Many photon therapy clinics have adopted these methods (18, 19), while others have developed their own variations (20). However, knowledge of the safety of emerging radiation therapy technologies is inherently incomplete. Furthermore, emerging technologies have been identified as a common source of delivery errors (21). It is not yet clear how best to address the safety of emerging technologies, particularly for systems that treat moving tumors. It has been suggested that developing and simultaneously performing quality assurance during clinical trials of emerging technologies decreases safety errors (22). Prospective analyses, such as FMEA, could be a useful tool for emerging technologies (23), including conformal ion therapy for treating moving tumors (24). Though several motion handling strategies with ion beams exist (25–28), few of these motion handling strategies are integrated into their beam delivery systems (29). Relatively less is known about the safety risks of a modular motion-synchronized dose delivery system (M-DDS) for ion beam therapy (30), and no comprehensive assessment of the safety of a dose delivery system with integrated motion compensation has been reported in the scientific literature.

The purpose of this study was to apply an established method to analyze safety risks from a novel, modular, motion-synchronized dose delivery system for scanned ion beams. The system is in the late stages of preclinical development and testing. Here, we focused on the beam delivery process, identified motion-related errors, and implemented corresponding solutions. The performance of the M-DDS has been previously described by Lis et al. (2020). We developed and performed sample commissioning-style tests and quality assurance (QA) tests. These results provide information for subsequent clinical safety assessments of a novel, modular motion-synchronized dose delivery system in development.

## Materials and Methods

This work describes a safety assessment of a dose delivery system (DDS) that is undergoing pre-clinical testing at two ion therapy centers. The M-DDS is an extended version of a clinical products used at the National Center for Oncological Hadrontherapy (CNAO) and MedAustron that have undergone full safety certification. The two most important extensions to the DDS were to make it portable and to allow for motion mitigation by synchronizing beam delivery to anatomical motion. The performance of the prototype motion-mitigation DDS was previously demonstrated, including preliminary tests such as the delivery of conformal, motion-synchronized beams (30). These results focused on proof of concept and the preliminary characterization of performance, but not safety. However, failures in the functionality of the M-DDS components could theoretically compromise patient safety. To minimize this risk, safety was integrated into each stage of development, in an effort to maintain the existing safety system for reintegration of the M-DDS into CNAO.

The assessment strategy described in this work applies an established methodology from AAPM Task Group 100 (13). This strategy is a prospective risk analysis method that has been widely used in the medical and other industries. With this strategy, we first defined each step of the treatment process, in detail, with a process map. We then identified possible errors that could occur at each step and quantified the effect on patient treatment with an FMEA. Finally, we identified the causes of errors with a fault tree analysis (FTA). After determining the potential safety risks, we developed and tested solutions for these errors (13).

The prospective risk analysis was performed on a DDS, with integrated capabilities for target motion compensation (30). For convenience, the general characteristics of the M-DDS are summarized here. The DDS was adapted from the DDS found at CNAO to function with the therapy research line (Cave M) at GSI Helmholtz Center for Heavy Ion Research (GSI). The DDS is composed of commercial field programmable gate arrays (FPGAs) (PXIe-1085; National Instruments, Austin, TX), which control each beam delivery component, including the scanning magnets, beam monitoring detectors, timing system, and interlock system. The DDS has been modified to synchronize the delivery of 4D-optimized plan libraries (24, 31) to detected target motion.

Motion mitigation features are integrated into the M-DDS. The first step of motion synchronized dose delivery is creating 4D treatment plan libraries from 4DCTs, where each plan in the library contains delivery information for a motion phase within the 4DCT. During beam delivery, a motion-monitoring device continuously monitors the tumor position, from which the M-DDS determines the current motion phase (32, 33). The delivery progresses in sequence until the tumor position has entered into another motion phase and delivery is redirected to the plan from the plan library that corresponds to the detected motion phase. Further information on the M-DDS is described by Lis et al. (30).

Good manufacturing practices were followed through the development of the M-DDS. Critical processes in the M-DDS were maintained from the clinical M-DDS design and all changes were evaluated experimentally in the clinical environment. The implemented software design choices were based on the existing software structure, so most uncovered sources of error were found to be related to unclear workflow and limitations to memory or signal speed. All changes and additions were documented. In the following sections, we describe a safety assessment strategy for the M-DDS. Additionally, we provide example safety and quality assurance tests for the M-DDS.

### Process Steps

The first step of the prospective risk analysis was to identify the sub-processes that occur through the course of treatment with a process map. A process map is a visual representation of a process that demonstrates the flow of each step from start to end. We divided the process of treating a patient with scanned ion beams into 10 main stages, based on the guidelines proposed by the World Health Organization (34). In this study, we focused on six of these stages—planning, simulation, patient setup, treatment delivery, treatment verification, and monitoring—as these were the most relevant to treating moving targets. For each of these stages, we identified the sub-processes that occur at an ion beam therapy center (21), such as delivery of an iso-energy slice (IES) during the course of treatment. We then amended each stage to include any additional sub-processes when delivering motion-synchronized ion beams with the M-DDS, such as redirecting the plan delivery to compensate for detected motion. Modes of failure were then identified for each of these sub-processes.

### Failure Modes and Effects Analysis

The FMEA assesses the likelihood and impact of failures from each step of a process. An FMEA was applied to each of the identified process steps and potential modes of failure at each step were described. Each of the failures were assigned a rank value on a numerical scale of 1 to 10 for each of three safety indices: the severity index (S) is the extent of harm of the failure on the patient, the occurrence index (O) is the likelihood that a cause will result in a failure, and the detection index (D) is the likelihood that a failure will not be detected. All three of these indices are estimated under the assumption that there was no safety check in place for that failure. The corresponding definitions for the rankings of each of these values are summarized in **Table 1**. This data has been adapted from the TG-100 (13). The failures were then ranked by calculating the risk priority numbers (RPN), which is the product of these three indices (RPN = S × O × D). RPN values of above 125 were considered high risk, and any S score above 5 was also considered high risk. One example error is the gradual drift of the scanning magnet throughout the course of delivering an IES. This could potentially cause limited toxicities or overdose, as the drift may be on the scale of a few mm, and would potentially occur for every delivery in the absence of position feedback. Such an error would be difficult to detect without monitoring. The resulting RPN would then be 6 × 10 × 7 = 420. Selected FMEA indices were agreed on by a consensus group of experts, including the authors on this work.

**Table 1:** Numerical scale used to assign rank values to Severity Index, Occurrence Index, and Detectability Index for each failure.

Rank value	Severity Index	Occurrence Index (mean time between failure)	Detectability Index
1	No effect on patient care	+ 4 years	Impossible to miss
2	Inconvenience or delay in care	2 years	“
3	“	1	Highly likely to notice
4	Minor dosimetric error	6 months	Easy to detect
5	“	1 month	Fairly easy to detect
6	Limited toxicity or overdose	2 weeks	Fairly difficult to detect
7	Potentially serious under- or overdose or toxicity	1 week	“
8	“	3 days	Nearly undetectable
9	Very serious under- or overdose or toxicity	1 day	“
10	Patient death	1 hour	Undetectable

These data have been adapted from the TG-100 report on failure modes and effects analysis (FMEA). For each case, a rank of 1 was considered harmless, and a rank of 10 was catastrophic. Chosen rank values were based on observed or estimated.

### Fault Tree Analysis

Causes of each identified failure were mapped out with a fault tree analysis (FTA). The FTA allows for visualizing potential points to perform quality management procedures and to minimize the propagation of errors (35). Each failure mode was traced back to its potential causes using a logic tree. Possible failure modes include user errors, such as selecting the wrong delivery setting or incorrect patient setup, software errors, equipment failures, and patient non-compliance. For the example of an error in delivery of an IES, the cause could be traced back to a faulty position feedback from the beamline monitors to correct the scanning magnet power supplies. Other causes of the error could also be postulated, including slow scan speeds and incorrect magnet calibration. Once causes for each failure were identified, methods to eliminate the cause or to detect the possibility of each failure were developed.

### Solutions and Tests

After identifying the potential solutions for the safety risks, appropriate solutions were implemented and error-handling tests were designed and performed. Solutions for failure modes can be classified into several categories: permanent corrective actions, error states and interlocks, commissioning and quality assurance tests, and plan verifications. Permanent corrective actions are changes in the workflow of the planning or delivery software or user protocols in order to eliminate the possibility of that failure mode occurring. This can include implementing redundancies, such as redundant devices and communication protocols. Pass-fail tests were performed by simulating error states and checking that the delivery system entered an error state or triggered an interlock. Commissioning and QA tests are tests that verify that the system consistently works according to manufacturer specifications and within acceptable fault tolerances. Plan verification tests verify the accuracy of patient plans. For example, scan magnet position errors can be prevented in several redundant ways. Two position-monitoring chambers are used during delivery to monitor the accurate delivery of beam spots within an IES. Additionally, interlocks are in place in the case of failure of one of the monitoring or scanning magnets. Finally, daily QA is performed to confirm the functionality of the entire delivery system. Whenever possible, permanent corrective actions were implemented.

#### Description of Error Handling Tests

Error handling tests were created for each of the failures identified in the FTA. Pass-fail test cases were written for each of these failures. Corresponding software tests were then created that inject error scenarios into the delivery process to confirm that the DDS can respond to the respective error. In the case that an error-handling test failed, the underlying delivery software was modified to prevent the error from occurring. In other cases, an interlock was also implemented to trigger the interruption or termination of treatment in the presence of a motion synchronization error. The implemented interlocks were also tested by injecting error scenarios into the delivery process.

#### Daily, Weekly, and Annual QA

The performance of the accelerator and beam delivery components was characterized through a series of quality assurance measurements. While existing QA protocols (11) will confirm the functionality of most aspects of motion-synchronized dose delivery, additional tests must still be performed to ensure the performance of additional features, including the motion monitoring system and 4D plan library. QA tests were designed that prioritized a simple set up, are multi-purpose, are fast and use well-characterized phantoms. A QA concept was designed to test the safety and reproducibility of motion-deliveries. Where possible, the current clinical protocols at ion therapy centers, such as those used at CNAO (36) were extended to include motion scenarios. Each test was verified experimentally in a clinical setting at CNAO.

Daily QA tests were designed and performed for measuring field uniformity, beam spot positions, and beam reproducibility. Setups with water-equivalent plastics (RW3 slab phantom; PTW, Freiburg, Germany) and radiochromic films (EBT3 Gafchromic; International Specialty Products, Wayne, NJ) were selected, as their assembly time is fast and they are both well-characterized systems. The daily QA procedures found at CNAO were modified and applied to test the delivery quality of motion compensation with the M-DDS. This allowed faster delivery that was not dependent on additional custom-made software for analysis. The daily QA setup, a radiochromic film, mounted behind 2 cm of water-equivalent plastic, was mounted on top of a linear stage with programmable motion patterns (M-414.2PD; Physik Instrumente GmbH, Karlsruhe, Germany). Motion amplitudes, detected from an optical laser distance sensor (OD100 − 35P840; SICK, Waldkirch, Germany), were converted into motion states (30). For each test, the clinical (non-moving) procedure was performed first, followed by the motion-compensation variant. For these beam deliveries, the linear stage moved with 20 mm amplitude sinusoidal or Lujan2 motion (37–40) while delivering a uniform square profile with eight surrounding spots. The purpose of each test and the measurement criteria are summarized in **Table 2**.

**Table 2:** Description of the purpose and pass criteria for each quality assurance test.

Test type	Quantity tested	Pass criteria
*Daily QA*		
Field uniformity	Homogeneity index (41)	≥95%
Spot shape	FWHM in X and Y direction across scan field (11)	Symmetrical
Spot position	Distance to agreement (42)	< ± 1 mm
Motion-monitoring system functionality	Function test	Functioning
*Weekly QA*		
Beam monitor calibration reproducibility	Coefficient of variation (43)	<1%
*Annual QA*		
Dose distribution with homogeneous phantom	Percent error from expected dose (36)	<5%
Dose distribution with heterogeneous phantom	Percent error from expected dose (36)	<5%
*QC*		
Motion-monitoring system performance	Distance to agreement (42)	<0.1 mm

All films were calibrated in a series of steps. Before the QA tests, standard dosimetry was performed (44), and calibration films, composed of eight squares with fluences from 2 × 10^5^ to 4 × 10^7^ particles/mm, were acquired for each batch of films. The calibration plan was delivered with 280 MeV/u carbon ions to films placed behind 2 cm of water-equivalent plastic. After delivery, the QA films were scanned with a laser film scanner (VIDAR DosimetryPRO Advantage Red; VIDAR System Corporation, Herndon, VA, USA) in landscape orientation, using 16 bit sampling and a 300 dpi resolution. A batch-specific optical density correction was applied to each film by subtracting out the optical density of an unirradiated area of the film, using an image analysis software (ImageJ version 1.52a; National Institute of Health, Bethesda, MD). The calibration films were used to apply a calibration curve, converting optical density to particle intensities. Each film was cropped, aligned, and corrected for linear energy transfer (LET) quenching effects (43), by applying a relative efficiency (RE) correction curve as follows:

$$RE\left(LET\right)=\frac{D_{280MeV/u}\left(netOD\right)}{D_{abs}}$$

where D_280MeV/u_(netOD) is the 280 MeV/u carbon ion dose needed to produce the measured, corrected optical density, and D_abs_ is the actual dose delivered with carbon ions to the film. After, calibration, the films were analyzed.

The uniformity was assessed through the homogeneity index (HI), which is defined as

$$HI=100-\;\frac{D_{max}-D_{min}}{D_{p}}$$

where *D_max_* and *D_min_* are the maximum and minimum measured absorbed dose, respectively, and *D_p_* is the prescribed dose. The *HI* was measured in the center 70% of the target volume. *HI* values above 90% were considered clinically acceptable. Additionally, the beam spot position accuracy was assessed by measuring the relative distance between each pair of beam spots in 2D profiles of the films. The beam spot shape and distortion was measured by determining the FWHM in the horizontal and vertical directions. Beam reproducibility tests were performed by comparing the delivery results across several weeks with a coefficient of variation (45). Finally, the increases to QA delivery time were assessed by measuring the setup and delivery time for each test and estimating the increased time for QA, when performing motion-specific testing alongside the currently performed QA tests in each treatment room.

Equipment quality control (QC) is generally also performed daily to verify the functionality and accuracy of treatment equipment. To verify the performance accuracy of the motion-monitoring equipment (a linear stage and an optical distance (OD) laser) the linear stage was programmed to move in an increasing stepwise motion pattern. The measured OD laser signal was compared to the motion files for the linear stage.

Annual QA is a series of extensive tests to measure machine performance. Annual QA tests were performed to measure dose distributions with a 3D homogenous setup and a 3D inhomogeneous setup. First, 4DCTs of an heterogenous phantom (CIRS 062 electron density phantom; CIRS, Norfolk, VA, USA) and a water tank (MP3-P; PTW, Freiburg, Germany) were acquired. 60 × 60 × 60 mm^3^ cubes were delivered to 12 small-sized ionization chambers (PinPoint 3D ion chamber model T31015; PTW, Freiburg, Germany) in a water tank for both setups. For the 3D inhomogeneous setup, density compensation measurements were performed by the heterogeneous phantom mounted in front of the water tank. Both deliveries were performed without motion, and with motion compensation. Standard deviations for measured doses of < 5% were considered acceptable.

#### Patient Plan Verification

Plan verification, or patient specific quality assurance (PSQA), is performed to assure the accuracy of a treatment plan. Treatment planning and treatment delivery errors, unique to the motion mitigation system, can be discovered through PSQA testing. Errors may include selecting the wrong motion trajectories during planning or delivery, or planning with an unintended motion compensation strategy. If not detected, these errors could lead to severe dose degradations. Several plan verification methods were chosen to test the extent to which planning and delivery errors related to mitigating for respiratory motion can be detected. The chosen PSQA tests included patient verification protocols that are used in ion therapy clinics, including 1) 3D dose measurements with small-sized ICs, 2) repeat 2D measurements at three depths with a 2D ionization chamber array detector (Octavius 1500 XDR; PTW, Freiburg, Germany), and 3) log file analysis. Additionally, 3D dose measurements were also performed with 4) a stack of radiochromic films (46). These measurements were analyzed with standard deviation calculations, gamma index analysis, and through the evaluation of dose volume histogram (DVH) metrics, respectively. To perform these measurements, the detector or film was placed into its respective holder, and the holder was mounted onto a linear stage. 4D-optimized patient plan libraries were delivered to each detector setup. The linear stage was programmed to move with trajectories derived from the patient 4DCTs. Other aspects of PSQA were also considered when designing each setup, including favoring simpler phantom setup processes and higher resolution of the recorded data.

**Figure 1:**
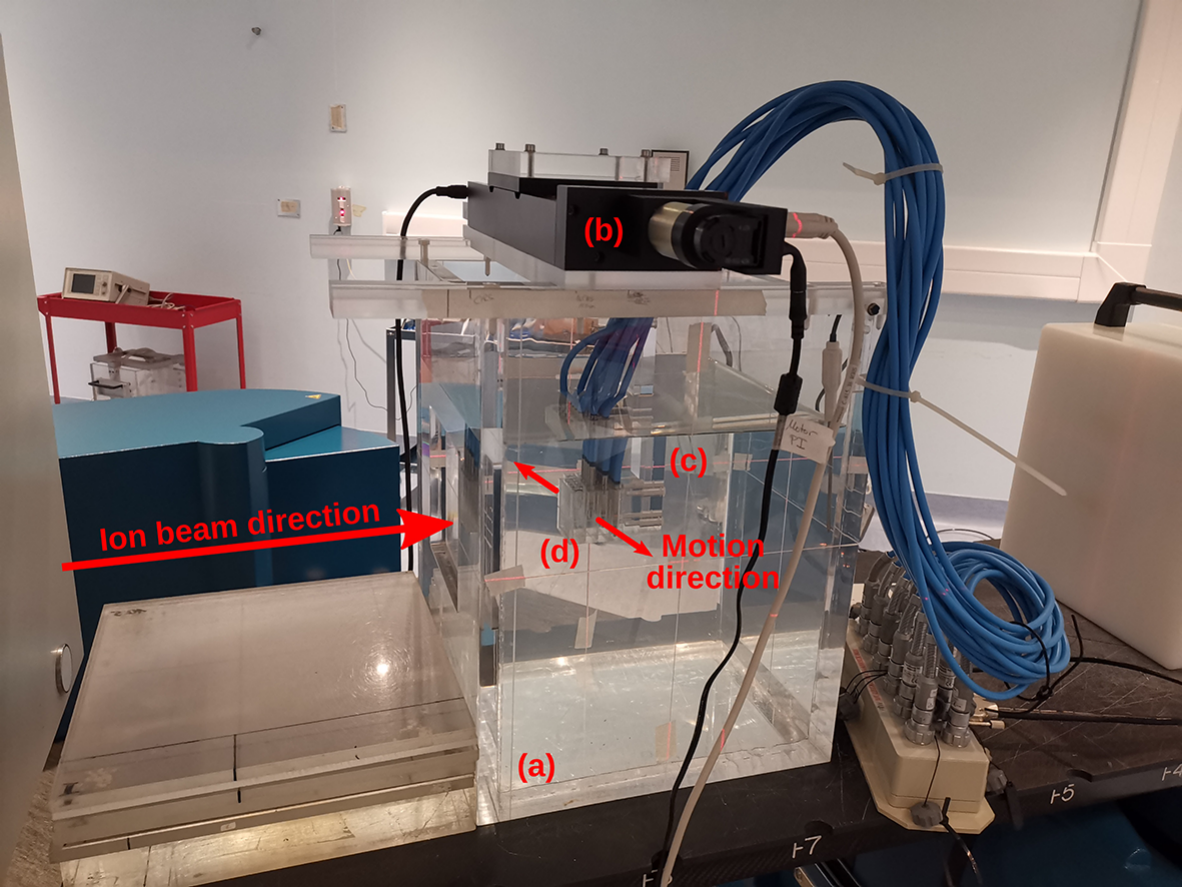
Experimental setup for patient-specific quality assurance (PSQA) measurements with **(A)** a water tank and **(B)** a linear stage mounted on top. The linear stage is programmed to move a variety of attachments in periodic, respiratory-like motion patterns. Here, **(C)** a holder with **(D)** 12 small-sized ionization chambers (IC) inserted inside is attached. This IC holder aligns with the isocenter markings on the water tank phantom, which is filled with water.

3D dose measurements were performed by delivering patient plans to 12 small-sized ICs. These ICs were selected, as they are used in several clinical ion facilities for patient verification (47–49). The ICs were inserted into a custom removable holder, connected to a linear stage that generated the motion of the ICs (**Figure 1**). The linear stage was mounted on top of a water tank, similar to the commercially used water tank phantoms, and the water tank was filled with water. Each patient plan was delivered to this phantom, with the linear stage moving with the patient’s breathing pattern. Each IC recorded a dose measurement and standard deviations were calculated from these doses. The chosen prescription dose was 6 Gy per fraction. Standard deviations of < 5% are typically considered acceptable.

**Figure 2:**
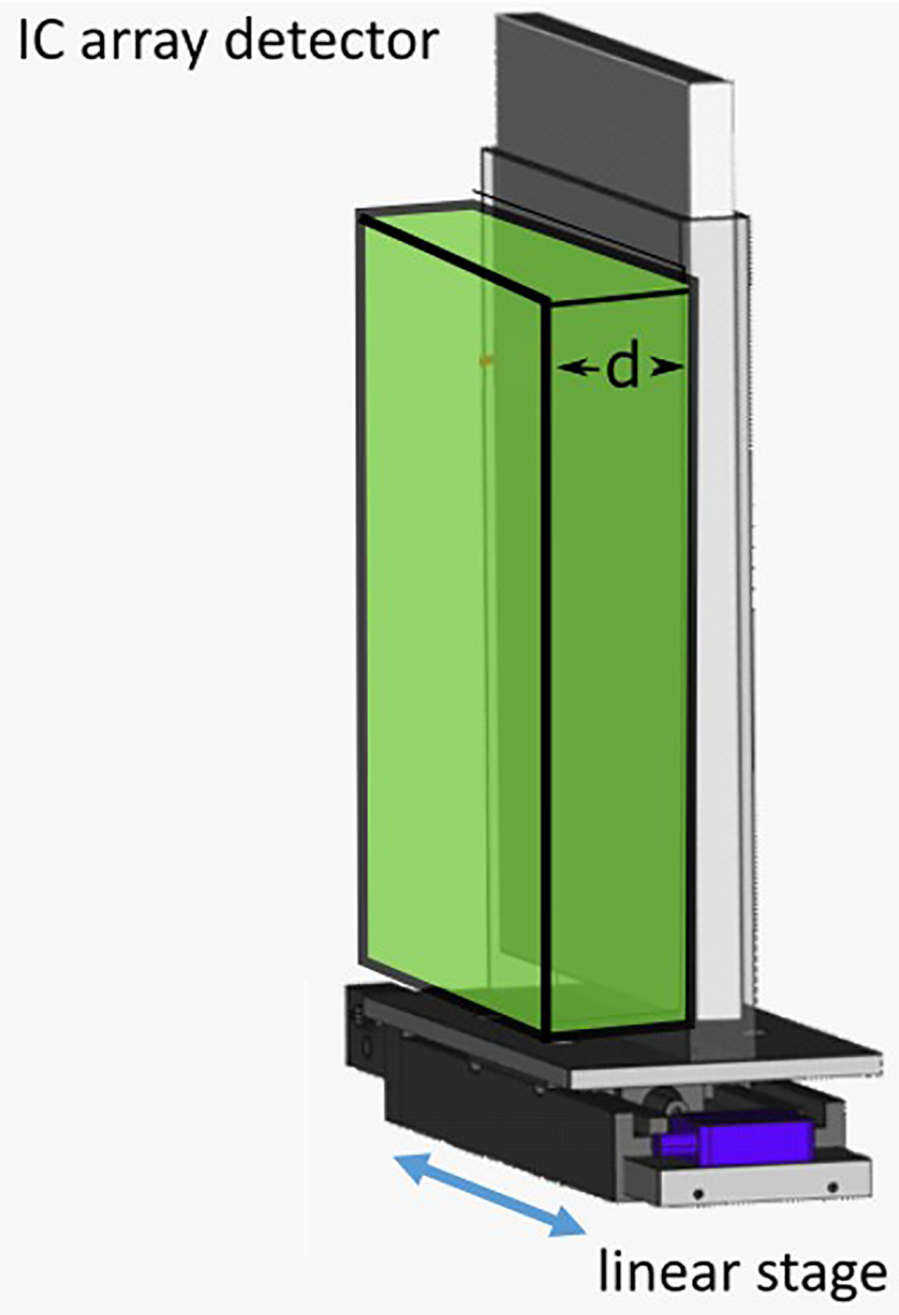
Schematic of the ionization chambers (IC) detector array setup. The IC array detector is placed inside of a 5 mm polymethylmethacrylate (PMMA) holder, mounted onto a linear stage. Water-equivalent plastic of thickness “d,” corresponding to the distal, middle or proximal depths of a target, are then placed in front of the detector.

2D dose measurements were performed by delivering the patient plan to a 2D ionization chamber array detector at three tumor depths. The chosen depths corresponded to the proximal end, middle, and distal end of the tumor depth. The appropriate thickness of water equivalent plastic (RW3; LAP GmbH, Lüneburg, Germany) was placed in front of the detector for each case (**Figure 2**). This setup was mounted on the linear stage to generate patient motion and a patient plan was delivered to the detector with and without motion. The delivery results were compared to the planned doses for each depth with the gamma index analysis (42), where a criterion of 3%/3 mm was used, with a dose threshold of 5% of the prescription dose. Pass rates of >90% for measurements made at all three depths were considered passing.

**Figure 3:**
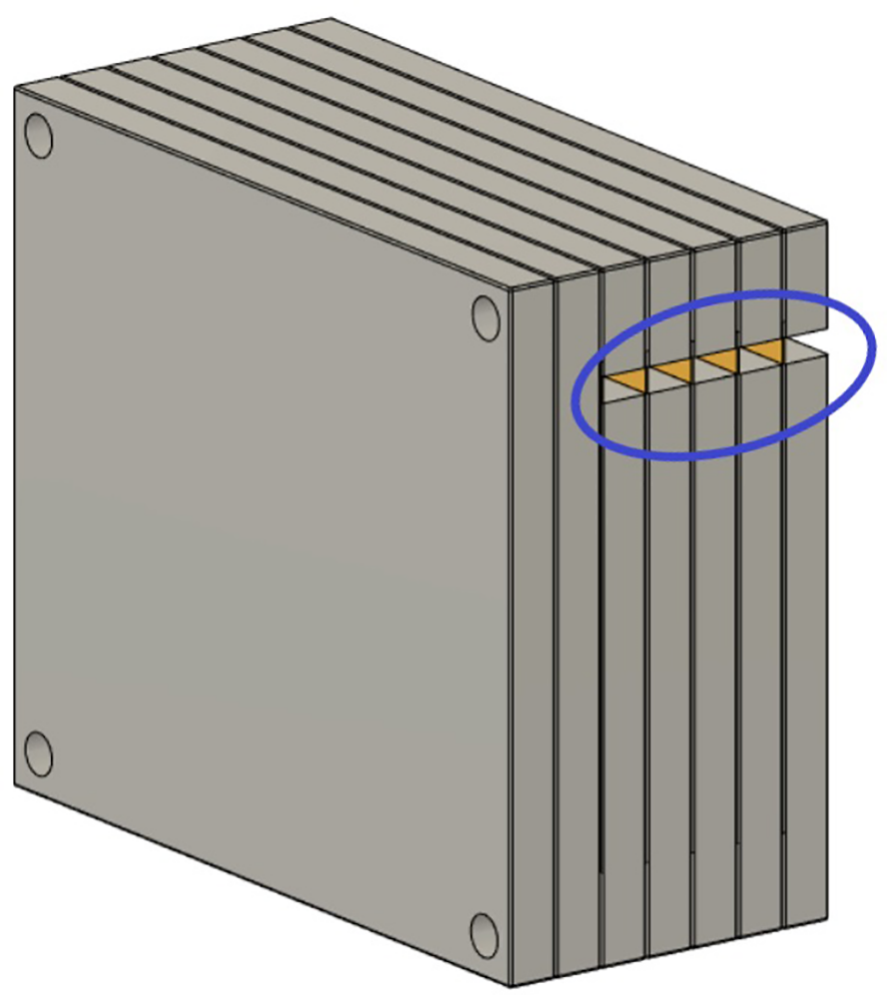
Schematic of the film stack. The film stack contains up to nine slabs of polymethylmethacrylate (PMMA) with precisely machined slots for holding radiochromic films (5” × 4”) as well as several slabs of additional PMMA to vary the delivery depth. The phantom measures 15 × 13 × 9 cm^3^ when nine slabs are in place. The slabs are aligned and held together by plastic screws at each corner of the phantom. The lateral cutouts (blue circle) are used for easy access of the irradiated films (yellow) without the need to disassemble the film stack phantom.

3D measurements with a stack of films were made to acquire higher resolution dose distributions. Seven 5” × 4” radiochromic films were slotted into an in-house built film holder phantom. The phantom was composed of a stack of 15 × 13 × 1 cm^3^ polymethyl methacrylate (PMMA) plastic slabs, with slits for the films, as seen in the technical drawing in **Figure 3**. This setup was mounted on top of the linear stage, which generated periodic motion. Each film was numbered and labeled at the top right corner before delivery. Each patient plan was delivered to the film stacks, in the presence and absence of motion. After delivery, the films were processed as described in section above. Each film was then analyzed with the gamma index analysis. The average gamma index pass rates for each film stack were evaluated using an in-house developed research software for data analysis, ArrayParser, where each film was compared to the respective treatment plan at the appropriate tumor depth. Gamma index pass rates of above 90% were considered clinically acceptable.

Log file analysis has been used to decrease PSQA measurement time. The patient plan was delivered to the IC array detector and log files from the scanner magnets and nozzle detectors were used to reconstruct the delivered doses on the planning 4DCT, using TRiP4D (30). The motion signal from the linear stage was used directing plan delivery during motion-compensation. However, a simulated motion signal could also be used for these measurements. The dose reconstructions were then compared with planned dose distributions using the gamma index analysis to a criterion of 3%/3 mm, where > 90% pass rates were considered acceptable.

## Results

### Process Map

**Figure 4:**
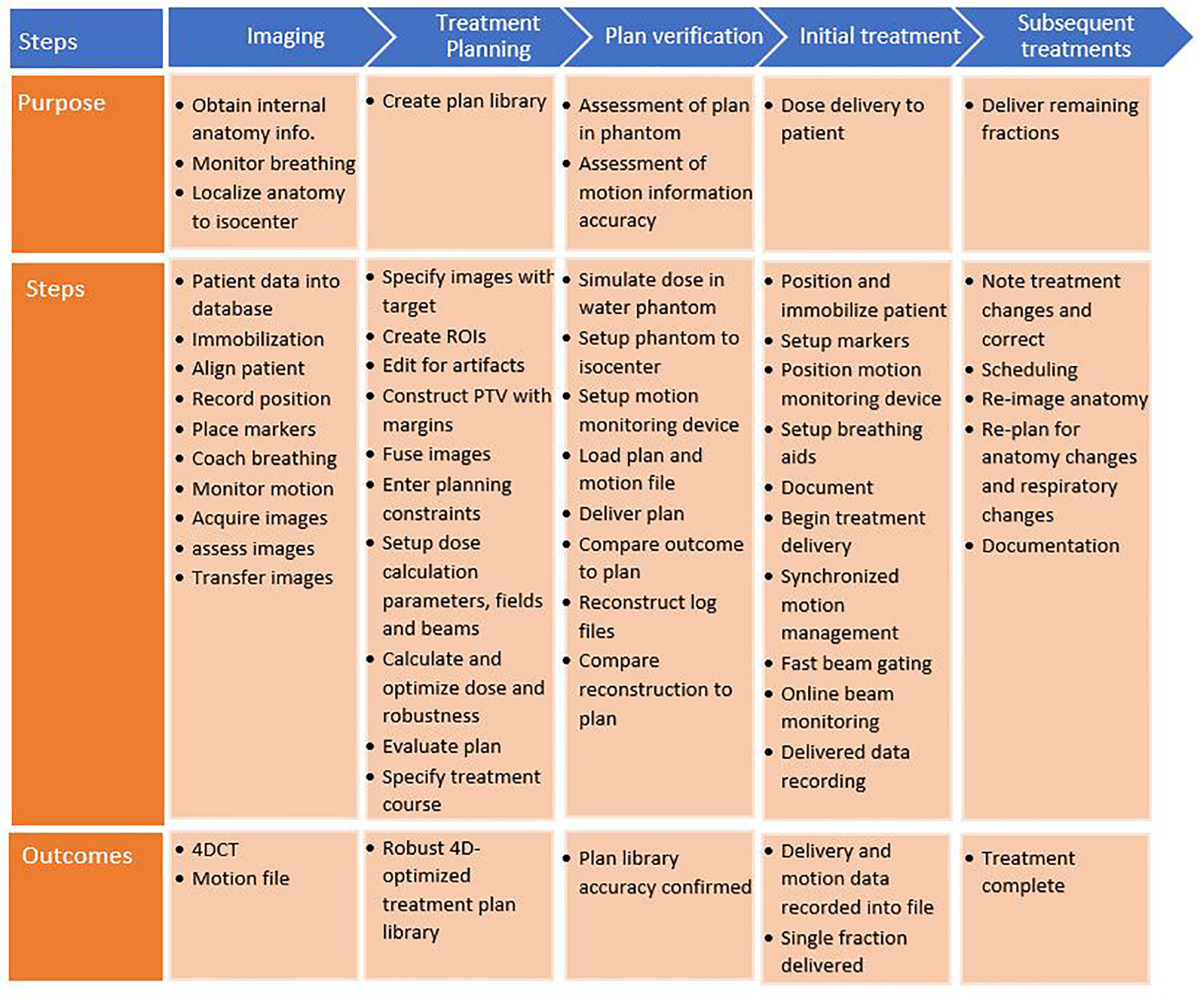
Process map for motion-synchronized dose delivery as commonly found in ion therapy clinics. The treatment process is broken down into five categories: imaging, treatment planning, plan verification, initial treatment fraction, and subsequent treatments. Each step in these processes is described.

A process map was created to map out the sub-processes of patient treatment at a typical ion therapy center. The processes for moving tumors are presented in **Figure 4**. This map consists of five main workflow steps, starting with patient imaging through the last fraction of treatment delivery. Patient-specific verification procedures were included as well. Several of these steps were critical to patient errors.

### Failure Modes and Effects Analysis

A systematic evaluation of the process map identified 58 failures. A subset of these failures is shown in **Table 3**, including the highest ranked failures during treatment delivery. Values for S, O, and D indices were estimated based on the metrics from

**Table 3:** Summary of failure modes and effects analysis (FMEA) results for potential errors during patient therapy with motion-synchronized ion beams using a DDS with integrated motion compensation controls. Risk priority numbers (RPNs) of over 125 were considered high risk.

Failure mode	Severity	Occurrence	Detectability	RPN
Patient movement	7	6	7	294
Absolute change in breathing position	6	7	7	294
Patient alignment	7	5	6	210
No gating during sporadic movements	5	5	8	200
Gating window too large	6	8	4	192
Beam sweeping distance dose	3	8	9	192
Sending incorrect motion phase info	7	3	7	147
Error creating of slice files	7	3	7	147
Failure to gate	9	2	8	144
Position calibration incorrect	8	2	7	112
Changes to respiration pattern	7	8	2	112
Setup of motion management device to wrong position	9	2	6	108
Patient not re-imaged after anatomy change	7	3	5	105
Position feedback missing/not working	4	5	5	100
Determined wrong number of motion phases	6	2	5	90
Incorrect motion direction	9	2	5	90
Loading wrong treatment plan	4	2	10	80
Motion data recording stops	4	2	9	72
Failure to progress to next slice	6	2	5	60
Plan incomplete	6	2	5	60
Motion signal loss	9	2	3	54

**Table 1** and were used as a guide to determine the highest risks for conformal, motion-synchronized therapy. All failures with an RPN of 125 or greater and all failures with a severity > 5 were considered in this study. In total, 41% of failures were identified to be low risk (RPN = 1–75), 33% were found to be medium risk (RPN = 76–125) and 26% were found to be high risk (RPN > 125). The highest ranked failures, with an RPN score of 294 were caused by delivery errors due to patient movement and absolute changes to breathing position. Potential failures that are common to both static site and moving site treatments were not included in the analysis, such as miscommunications between physicians and technicians and certain treatment planning errors.

### Fault Tree Analysis

**Figure 5:**
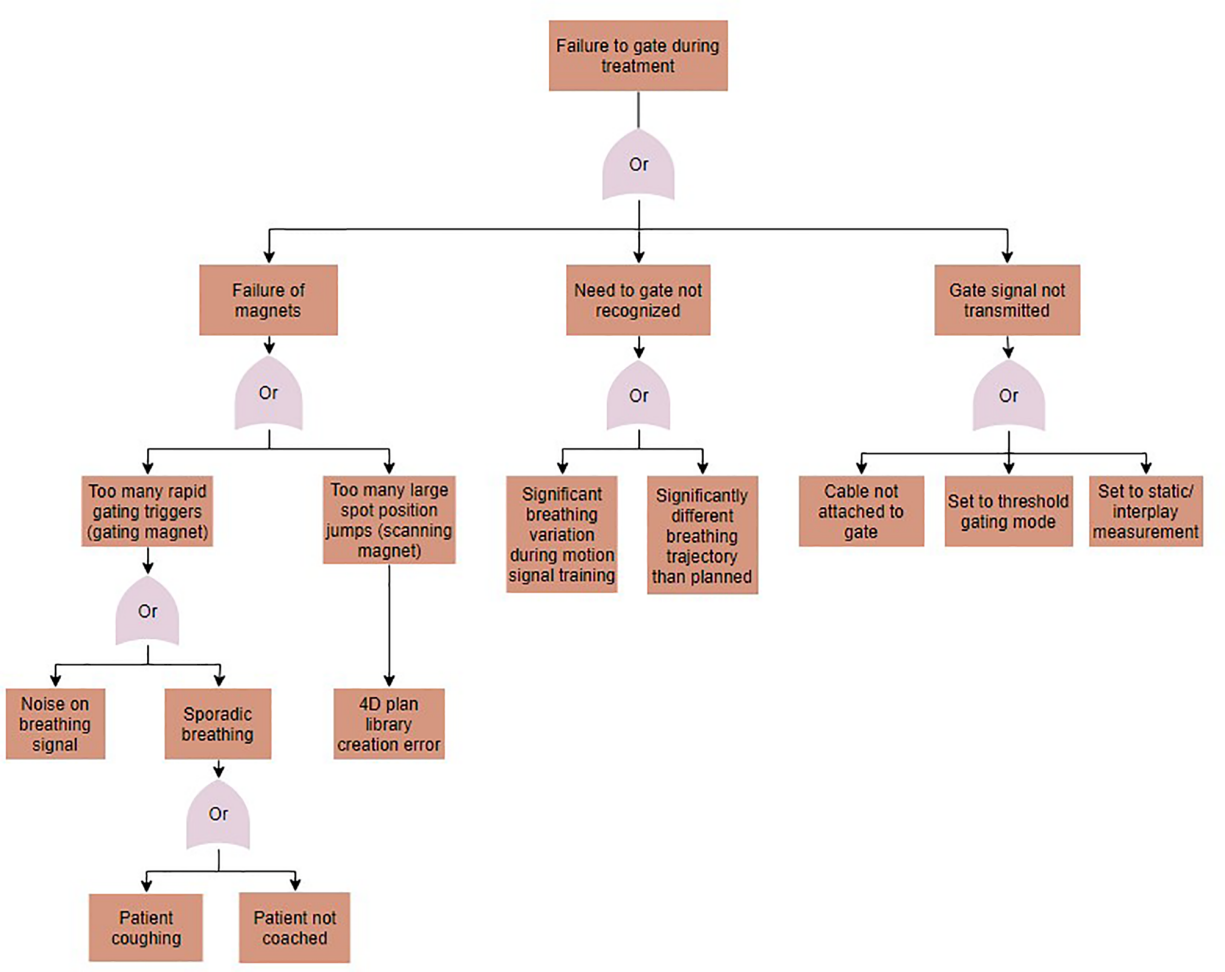
A fault tree analysis for the case of gating failures during motion-synchronized dose deliveries.

Fault tree analysis was performed to identify sources for the potential errors identified in the FMEA. Solutions for each error in the FMEA were identified, implemented, and tested. A sample fault tree can be seen in **Figure 5** for the case of gating magnet failure. Identified causes of error included human error, such as setting the treatment to the wrong delivery mode, machine errors, such as noise on the motion signal, and treatment errors, such as changes to the breathing patterns between image acquisition and treatment. Proposed solutions for these errors varied for each error type, and included disabling the option for gating when a 4D plan library is loaded, implementing a noise reduction filter on the motion signal, and using a monitoring method that compares planned to measured motion and gates delivery when out of range. Following implementation, the solutions to the identified errors were tested.

### Safety Testing

Solutions were implemented to prevent the identified errors from occurring and each solution was tested. The implemented permanent corrective actions included noise filtering to smoothen the respiratory signal, automatic extraction and setting of the number of motion phases from the treatment plan library, and implementing a checkpoint to prevent the beginning of delivery until the motion-monitoring system is calibrated and sending a motion phase. Many errors were identified to be due to mistakes made by the user. Some examples include setting up the motion-monitoring system in an orientation other than what was used during planning image acquisition, and not accounting for changes to the tumor position, relative to isocenter (50). In these cases, the proposed solutions included performing a second check by another clinician or reimaging the patient periodically. Additionally, using a checklist to check the patient setup before treatment could reduce the incidence of user errors.

#### Pass Fail Tests

A series of error handling tests were created to test each of the implemented solutions. These tests included pass-fail tests, where the expected results included either triggering a temporary interlock, aborting treatment, or entering the “setup error” state. The summary of pass-fail test results is listed in **Table 4**.

**Table 4:** Summary of pass-fail tests and results.

Error	Expected action	Result
*Incorrect plan library structure*		
Wrong number of beam spots in plan library	Setup error state	Passed
Missing motion information in plan header	“	“
Particle numbers below or above limitations	“	“
Plan library larger than size limitations	“	“
*Beam delivery errors*		
Motion signal lost	Beam aborted	“
Motion trajectory deviating from expected trajectory	Temporary gate	“
Scanning magnet failure	Interlock	“
Gating magnet failure	“	“
Delivery of a beam spot skipped	Treatment is halted	“
MMD file recording error	Treatment stops, errors message, and file dump	“
Treatment stops prematurely	Delivery data recorded	“
Motion calibration incomplete before delivery starts	Beam gate activated	“
*Treatment setup errors*		
Wrong motion compensation strategy selected	Set automatically from plan	“
Motion system not fully set up or not on	Delivery cannot start	“

#### Daily, Monthly, Annual QA

QA tests were proposed for identifying errors in the functionality of the motion-synchronized delivery components. These tests are summarized in **Table 2**. QA setups were chosen that are available or are similar to those found in ion therapy centers. Delivered profiles were analyzed for uniformity, agreement with the treatment plan, and for beam reproducibility, the uniformity index, and gamma index analysis were chosen as analysis metrics.

Delivery uniformity with motion-compensation was determined by assessing a square profile for a single energy of 240 MeV/u, delivered with motion-compensation, to a radiochromic film. HI for the static and 10 phase compensation deliveries were 95.3 and 94.8%, respectively. The spot position accuracy was found to be within ± 0.5 mm from the expected position. The beam spot shape was determined through a measurement of the FWHM in the X- and Y-directions, where the X-direction was the direction of motion. For static deliveries, this was found to be 3.9 and 3.4 mm, respectively, and for the 10 phase deliveries, this was found to be 4.7 and 3.3 mm, respectively. The observed broadening of the beam in motion direction is an indication of the residual motion both within and between the motion phases, and spot size increases of up to ± 0.5 mm were tolerated.

The increased time to perform both static and motion compensation QA tests was measured and compared to static QA alone. For the current clinical QA procedures, setup and irradiation time was found to be 5 and 3 min, respectively, and for the motion compensation QA, the setup, and irradiation time was found to be 5 and 10 min, respectively. The estimated increase in QA-personnel time for a facility with three treatment rooms is 3 × 7 = 21 min, based on current QA methods and experience.

Annual QA tests included measuring 3D uniformity in a homogeneous and heterogeneous phantom. The static cube delivery measured a homogeneity of ± 1.2% and the motion compensated delivery measured a homogeneity of ± 3.5%. The static cube delivery of 10 Gy, through the heterogeneous phantom, measured a homogeneity of ± 2.1% and the motion compensated delivery measured a homogeneity of ± 2.3%.

**Figure 6:**
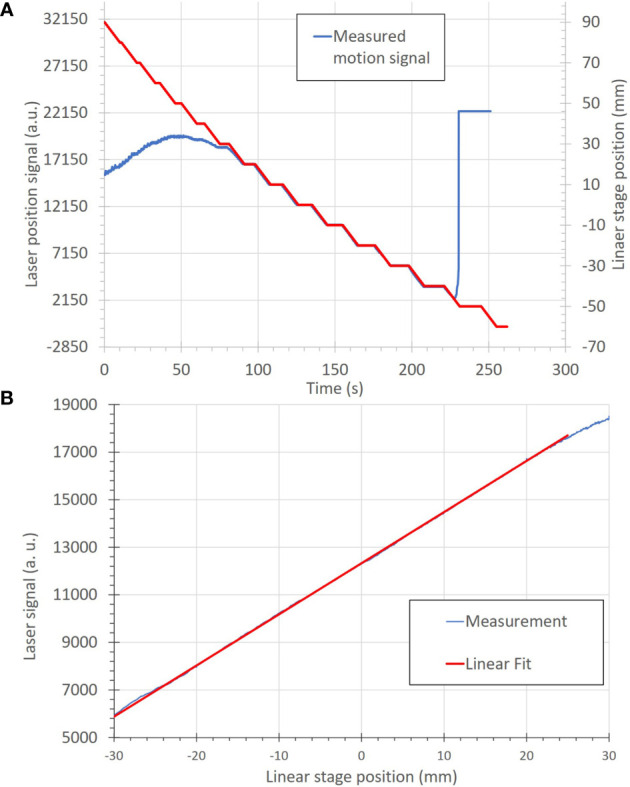
**(A)** Correlation of motion position to the measured signal of the motion-monitoring device (an optical distance laser sensor) for a step-wise motion pattern. The motion positions, in mm, (red) are uploaded onto the linear stage as a motion file and used to move the linear stage. The motion-monitoring device then records the relative displacement (blue) as an analog signal. The left and right portions of the motion signal show where the motion-monitoring device is out of signal range. **(B)** A plot of position accuracy between the optical distance laser signal (in arbitrary units) and the linear stage position (in mm).

The basic functions and accuracy of the motion-monitoring equipment were also determined. The measured motion was within ± 0.5 mm for all steps within the measurement range of −30 to 20 mm, as seen in **Figure 6A**. The agreement between the programmed linear stage positions and measured positions from the distance sensor are seen in **Figure 6B**, where the linear relationship indicates a high degree of measurement accuracy.

#### Patient Specific QA Results

Patient plan verification tests were performed to compare the measured accuracy of the delivered 4D plan libraries. Patient verification deliveries were found to be within clinical requirements; however, the measurement resolution varied for each approach. Results are summarized in **Table 5**. The small-sized IC measurements were found to be within ± 2.4 and ± 8.9% of the prescription dose for static deliveries and 10 phase motion compensation deliveries, respectively, where ± 5% is ideal, but due to residual motion, ± 10% was considered acceptable at this stage; however, higher precision may be necessary before clinical use. The measurements at three depths, with an ionization chamber array detector were assessed using the gamma index analysis. Pass rates (Pearson correlation score) for the static delivery were found to be 84.1% (0.9883), 100.0% (0.9947), and 99.4% (0.9983), for distal, middle, and proximal profiles, respectively. As only the distal measurement did not meet the pass criteria, log file analysis was performed to verify delivery quality. Pass rates (Pearson correlation score) for the motion-compensated delivery were found to be 91.6% (0.9901), 98.6% (0.9954), 90.9% (0.9971) for distal, middle and proximal profiles, respectively. The average gamma index pass rates for the film stacks were 92.4%, and 90.4% for conventional 4D optimized and robust 4D optimized deliveries, respectively. The average pass rate for the static delivery was 92.2%. The homogeneity for robustly optimized and conventionally optimized 10 phase motion compensated deliveries was 90.6% and 92.4%, respectively. Delivered dose data (DDD) log files were reconstructed and compared to planning simulations. The gamma pass rates were found to be 99.4 and 96.0% for static and motion-compensated deliveries, respectively. The measured motion signals were used to reconstruct the delivered beam spot position. The comparability of the data for each setup was limited by the lack of direct correlation between analysis methods (51). The small-sized IC and film stack phantoms were found to have a relatively fast setup and execution. Due to the limited number of detectors, small-sized IC measurements provided less information in cases where results were nearly passing. In those cases, DDD dose reconstructions were necessary to determine the dose distributions. 2D dose measurements with the IC array detector took multiple times longer to deliver than the film stack and small-sized IC measurements, due to the multiple measurements at varying depths.

**Table 5:** Summary of patient specific quality assurance results for four measurements.

QA test	Metric (pass criteria)		Static results	Motion mitigation results
Pinpoints	Dose deviation (± 5%)		± 2.4%	± 8.9%
Log files (planned to reconstructed)	Gamma index analysis (>90%)		99.4%	96.0%
Film stacks	Gamma index analysis (>90%)		92.4%	90.4%
IC detector to log file reconstructions	Gamma index analysis (>90%)	*Distal*	84.1% (0.9883)	91.6% (0.9901)
		*Middle*	100.0% (0.9947)	98.6% (0.9954)
		*Proximal*	99.4% (0.9983)	90.9% (0.9971)

These measurements included calculating 3D dose measurement agreement with 12 small-sized ionization chambers (IC), and calculating gamma index analysis pass rates for comparisons between log file reconstructions and treatment plans as well as IC array detector measurements. Acceptable criteria and analysis results are summarized.

Each PSQA method was also assessed for the ability to detect motion-specific planning and setup errors. Errors in positioning and orientation of the motion-monitoring system were visible for all PSQA methods, but were least apparent in the film stack deliveries. Selection of the wrong number of motion phases were only visible in log file reconstruction and films, but had little impact on the delivery results. Delivering a few beam spots to the wrong positions, not delivering a few beam spots and using the wrong motion file during planning were both only visible in the log file reconstructions, but there were no measurable changes to the treatment delivery quality. Finally, selecting the incorrect motion compensation strategy was visible on film stack images and IC detector array measurements but was not immediately clear without log file reconstructions. Log file reconstructions were quicker than other methods, due to requiring no phantom setup time. Likewise, log file analysis, and to some extent, film analysis, did not require precise positioning. IC array detector measurements took nearly three times as long as small-sized IC measurements, but provided a higher number of measurement points. In both cases, measurement analysis programs are available to assess plan accuracy. Films require additional time for digitizing and show a non-linear dose response. Calibrating the dose response of a film requires considerable effort before it can be used for QA measurements.

## Discussion

We have designed and tested a prospective risk analysis strategy for the motion-synchronized dose delivery system, developed for scanned ion beam therapy of moving targets. We created this strategy specifically to assess the safety of the motion mitigation portion of the DDS for its eventual use in the clinic. Additionally, we have implemented and tested solutions for possible errors related to motion mitigation. The major finding of this study is that we have identified the sources of and solutions to major errors with a comprehensive risk assessment strategy. We also obtained pre-clinical test results that suggest the clinical reliability in motion-synchronized dose delivery. The results of this study have determined that the proposed safety assessment tests can be utilized at ion therapy centers, which operate with the modular M-DDS.

The implication of this study is that the described comprehensive risk analysis strategy and proposed tests can serve as an example during initial safety, commissioning, and QA tests leading to implementation of the M-DDS into clinical use. This assessment is part of a larger effort to confirm and maintain the clinical safety of the M-DDS from the design stage through clinical implementation. The M-DDS has been implemented following good manufacturing practices, including testing at several stages and maintaining extensive documentation. The described preliminary safety tests suggest that the M-DDS is safe, reliable, and ready for additional tests, leading to eventually treating patients. Further, the proposed error tests and QA tests could be performed within clinically reasonable timeframes. The safety tests are not the final solution for commissioning and QA procedures within the clinic, but rather are an example of a general safety strategy for the M-DDS, which can be modified and extended to meet the specific needs of a particular clinic. Full acceptance testing and commissioning will be performed before re-implementing the modified DDS into the clinic.

This is the first implementation of a comprehensive, prospective safety assessment for pre-clinical testing of a motion-synchronized dose delivery system. The results of this study are coherent with the recommendations found in the TG-100 and other AAPM reports (2, 6, 11, 13, 52). All plan verification and QA tests were within clinical specifications. Log file analysis provided the additional benefit of recognizing individual beam spot errors and indicating other errors during treatment preparation that could otherwise be unnoticed and should be performed alone or along with regular plan verification.

Several studies have applied the safety assessment protocols presented in TG-100 to ion therapy (21, 53). To our knowledge, no studies have been performed to apply this approach to new technologies in ion therapy, including motion mitigation systems. Additionally, several studies have assessed the practicality of QA procedures. One study, by Hara et al. (54), describes a plan verification procedure for moving tumors. This strategy involves delivering patient plans to a 2D IC at three depths, and performing gamma analysis on each measurement. This study also concluded that this procedure is a beneficial QA procedure for moving tumors. Another study (55) described the process of plan verification with small-sized IC deliveries. The procedure and phantoms were modified in our study for motion compensation. Further, Matter et al. (56) investigated the capabilities of various plan verification procedures to ensure the integrity of treatment plans under a variety of planning errors. Of the measured errors, two cases were relevant considerations for motion-synchronized deliveries with the M-DDS: the “all spots shifted randomly” case, and the “increase in spot weights” case. In particular, residual motion within a motion phase can be as high as 1-2 mm, and is accounted for in planning margins. In contrast, small increases in spot weights may be possible when the beam is frequently gated or when there are frequent jumps in the scan position due to non-optimally created plans can result in non-trivial increases to the integral dose. As such, we conclude that log file analysis could provide a supplement to plan verification measurements. This study did not consider failures associated with using real-time imaging to monitor target motion. This is a vital part of motion-mitigation and will be investigated in future studies. Though a variety of imaging techniques and motion monitoring devices can be integrated with the M-DDS, additional risk analysis must be performed to identify and mitigate for failures associated with these devices.

Our study has several strengths. One is that this method is based on established methods (e.g., FMEA, FTA) that have been applied in clinics worldwide. It can be extended to any modular device with integrated motion mitigation strategies. Yet, it allows for identifying errors that may specifically occur when using motion synchronized delivery devices. Further, the strategy uses the official risk assessment proposed by the AAPM, can be applied to any motion-synchronized dose delivery implementation, and to any clinic that integrates the described M-DDS to their treatment systems. This strategy is a well-developed, well-known, and comprehensive risk analysis strategy. Though clinic-specific modifications will need to be made, the described approach can provide insight into potential complications, which could arise with the M-DDS. Finally, the presented quality assurance tests were designed with phantoms that are regularly found in proton and ion therapy clinics. These tests were performed at an ion therapy center (CNAO) under clinical conditions, with interlocks in place. QA and most PSQA tests were also performed at CNAO and GSI, except the film stack analysis, which was only performed at GSI.

One limitation of this study is that all safety procedures were tested with a predefined, 1D movement, generated by a motion-phantom. The motion patterns were well known and in complete agreement between the measured and actual motion. This is not a major limitation, as the delivery results with irregular motion will be characterized in future studies. Another limitation of this study is that no high-precision 3D dose distributions could be measured. 3D gels could potentially provide nearly instantaneous 3D dose distribution information. Gels produced by Maryanski et al. (57), which are readout with optical CT have recently been developed for 3D dosimetry of carbon ions. However, this strategy is still in the early stages of testing and has only been optimized for high doses for carbon ions. Another dosimeter for high precision 3D dose measurements would be measurements with a 2D IC array detector in a water tank phantom (58, 59). Though this strategy automatically provides high-resolution 3D dose information, this strategy requires delivering prohibitively long times for patient specific QA and assumes no phase dependence for motion-compensated deliveries; therefore, this method is better suited for beam commissioning. Another limitation of this study is that no independent dose calculations, such as Monte Carlo (MC) based dose calculations, were performed. However, this is not a major limitation, as MC dose calculations are typically time consuming; therefore, they are currently mainly performed to verify dose distributions when patient QA measurements do not pass. Additionally, the proposed patient verification methods are example solutions, and each clinic should select their appropriate plan verification method. Finally, MC performed at CNAO showed that MC simulations are sensitive to input conditions and simplifications to the MC models (60). Nevertheless, MC verification of patient plans has been growing in popularity and can serve as a powerful tool for independent dose calculation on well-characterized data sets.

The M-DDS is substantially complete and the current version has been transferred back to CNAO for use in the research room there. Additional features are still in development that aim to handle irregular respiratory scenarios and other complications related to respiration; these will be completed before the M-DDS is implemented for clinical use. Additionally, interoperability with other centers and full compatibility to DICOM and I-HERO standards will be implemented, along with any necessary regulatory approvals for human use, followed by a full clinical commissioning. Before clinical use, the plan library structure must also be implemented into commercial treatment planning systems.

## Conclusion

We have applied a comprehensive safety assessment strategy for the motion-synchronized portion of the dose delivery system. This work has shown that M-DDS is a clinically viable motion compensation strategy. The efficacy of possible QA procedures for motion-synchronized deliveries have been confirmed. Importantly, this strategy is specific to the motion-synchronized dose delivery system, but not to a specific clinic. Therefore, the presented methods can be adapted to other facilities using the M-DDS.

## Data Availability Statement

The original contributions presented in the study are included in the article/supplementary material. Further inquiries can be directed to the corresponding author.

## Author Contributions

ML and WN devised the topic of this work. CG and MDo devised the project. ML and MDo developed the M-DDS that is the device studied in this work. WN, CG, and MDu supervised this work. ML, MW, TS, and AP performed experiments, data gathering, and data analysis. ML wrote the manuscript with input from all of the authors. All authors contributed to the article and approved the submitted version.

## Funding

This project has received funding from the European Union’s Horizon 2020 research and innovation program under the Marie Sklodowska-Curie grant agreement No 675265, OMA—Optimization of Medical Accelerators.

## Conflict of Interest

The authors declare that the research was conducted in the absence of any commercial or financial relationships that could be construed as a potential conflict of interest.
